# Media Portrayal of Older Adults Across Five Canadian Disasters

**DOI:** 10.1177/00914150211024173

**Published:** 2021-06-21

**Authors:** Samantha A. Oostlander, Olivier Champagne-Poirier, Tracey L. O’Sullivan

**Affiliations:** 1 70363 Interdisciplinary School of Health Sciences, Faculty of Health Sciences, University of Ottawa, Canada; 2 200293 Department of Communications, Faculty of Humanities, University of Sherbrooke, Quebec, Canada

**Keywords:** media, discourse, older adults, ageism, disasters

## Abstract

We conducted a constructivist grounded theory approach in which discourse analysis was used to explore how Canadian news media portrays older adults and aging in a disaster context. We analyzed 119 articles covering five Canadian disasters and identified four themes: (a) stereotypes of older adults are presented on a positive–negative continuum in journalistic coverage of disasters, (b) journalistic coverage tends to exclude perspectives of older adults from relevant discourse, (c) journalists assess the value of losses for older adults—“home” as a central concept, and (d) disasters are framed as disrupting retirement ideals. A model was created to provide an overview of the journalistic coverage of older adults in disaster contexts. Understanding how old age and aging are presented by the media in a disaster context is important because it has further implications for informing and structuring disaster risk reduction policies.

## Introduction

Disasters are becoming more frequent and severe due to the negative impacts of climate change across the globe (United Nations Office for Disaster Risk Reduction [UNDRR], 2015). News media that communicate disaster-related information are influenced by the nature of the problem and how viewers interact with the content, as described by Downs (1972), in the context of the “issue-attention cycle.” Media coverage of disasters can influence the social construction of resilience and vulnerability, which can further influence disaster risk reduction (DRR) practices (Vasterman et al., 2005). This is particularly relevant with regard to high-risk populations—those who are at an increased risk of experiencing the negative impacts of a disaster due to the intersection of the social determinants of health (O’Sullivan & Bourgoin, 2010). Older adults, commonly defined as people age 60 years and older, are traditionally included in this high-risk category because extreme environmental conditions can exacerbate age-related illnesses and lead to increased vulnerability to negative outcomes (Bunker et al., 2016; O’Sullivan et al., 2014; UNDRR, 2015).

Research examining the media portrayal of older adults often focuses on age-related health conditions and comparison of older adults to other age demographics (Fraser et al., 2016; Rozanova et al., 2006). Negative depictions of aging and older adults are particularly evident in mass media (Fraser et al., 2020; Rozanova et al., 2006). Historically, researchers have identified under-representation and negative stereotyping as the two main issues in the media portrayal of older adults (O’Reilly, 1998; Vasil & Wass, 1993).

Recently, multiple types of ageism have been described, including intergenerational, intragenerational, and compassionate. Intergenerational ageism occurs when older adults are portrayed as unequal or inferior to younger demographics, whereas intragenerational ageism occurs when one group of older adults are viewed as unequal or inferior to a different group of older adults (Bytheway, 2005). Compassionate ageism is rooted in the idea that older adults are victims who need to be taken care of by others (Binstock, 1983, 2010). Rozanova et al. (2006) argue that the media often presents both negative and positive stereotypes related to aging, resulting in polarized ageism. All forms of ageism portrayed in the media are part of a larger cultural issue because the media help to co-construct our understanding of health and illness, and this is influential in the social construction of aging in general (Clarke et al., 2003; Fraser et al., 2016).

In this study, we examined how older adults are portrayed in news media covering disasters. While there are mixed opinions as to what constitutes a disaster, McFarlane and Norris (2006, p. 4) define a disaster as “a potentially traumatic event that is collectively experienced, has an acute onset, and is time-delimited.” In the context of disasters, there is relatively limited literature focused on older adults (Brown & Frahm, 2016). This is likely to change with the emergence of the COVID-19 global pandemic, which is disproportionately impacting the health and well-being of the older adult demographic (Centers for disease control and prevention [CDC], 2020). In this study, we focused specifically on natural disasters including, floods, tornados, and forest fires which, in recent years, have become common occurrences across the Canadian landscape.

We present a constructivist grounded theory approach in which a discourse analysis was conducted on news media coverage of five Canadian disasters: 2013 Alberta floods, 2016 Fort McMurray Wildfires, 2017 and 2019 Ottawa-Gatineau floods, and the 2018 Ottawa-Gatineau tornados. Our objective was to explore how Canadian news media portrays older adults and aging in a disaster context. Understanding how older adults and aging are presented by news media in a disaster context is important because it has further implications for informing and structuring DRR policies and practices.

## Methods

### Data Collection

We analyzed online news media articles covering five natural disasters from three Canadian provinces. To obtain relevant news articles, the database ProQuest and the websites CTV and CBC were searched using the name of each disaster: 2013 Alberta floods, 2016 Fort McMurray wildfires, 2017 and 2019 Ottawa-Gatineau floods, and 2018 Ottawa-Gatineau tornados. News articles—extracted and saved—met the following inclusion criteria: (a) mention any of the above disasters, and (b) written in English or French. Caption text and videos with no articles, articles with one line, and duplicates where at least 50% of the content matched a previous article were excluded.

When the dataset was complete, the articles were imported into NVivo12 software and the following search string was applied to screen all articles relevant to older adults: old; age; senior; elderly; retire; âge; vieille; vieux; personnes âgées; retraité. All articles were manually checked to ensure they were relevant to this study. Table 1 provides a summary of the data collection strategy.

**Table 1:** Data Collection Strategy.

Disaster	Words searched in ProQuest, CTV, CBC	Timeline (first and last article)	Number of articles extracted	Number of articles relevant to older adults/aging
2013 Southern Alberta Floods, AB	2013 Southern Alberta Flooding	June 20, 2013–June 20, 2014	597	19
2016 Fort McMurray Wildfires, AB	2016 Fort McMurray Wildfires	May 01, 2016–May 01, 2017	1132	22
2017 Ottawa-Gatineau Floods, ON & QC	Ottawa-Gatineau Floods; Ottawa Floods	April 01, 2017–April 01, 2018	310	19
2018 Ottawa-Gatineau Tornadoes, ON & QC	2018 Ottawa-Gatineau Tornado; Tornado Ottawa; Ottawa Tornado	Sept 21, 2018–Sept 21, 2019	339	32
2019 Ottawa-Gatineau Floods, ON & QC	2019 Ottawa-Gatineau Floods	April 2019–December 2019	287	27
**Total**			2665	119

### Data Analysis

The concept of ageism acted as a sensitizing concept guiding this study (Bowen, 2006; Charmaz, 2003). An inductive, constructivist grounded theory approach of the issue was generated by analyzing macro-level discourses related to the portrayal of older adults and aging in a disaster context (Charmaz, 2006, 2014; Foucault, 1972; Gee, 1999; Johnson, 2014). Throughout the analysis, efforts to suspend recourse to relevant literature were mobilized in an effort to remain open to the ways in which themes emerged and were identified from the data (Luckerhoff & Guillemette, 2011). Analysis of multiple and different types of natural disasters in this study supports the transferability of these results regardless of the nature of the disaster.

The first author (SO) engaged in familiarization of the data through re-reading, then inductively analyzed the data through the completion of open and axial coding, and selected 25 of the most relevant articles to submit to the other two authors. All three authors engaged in independent analysis to complete selective coding; the analytic process ended with a discussion, during which time different interpretations of the data were merged to produce a mutually agreed upon grounded theory of the issue. Nvivo12 software was used to facilitate the coding process.

## Results

The objective of this study was to explore how the Canadian disaster media portrays older adults and aging in a disaster context. Upon completion of our analysis, we identified four themes and created one model to provide an overview of the journalistic coverage of older adults in disaster contexts.

*Theme 1: Stereotypes of older adults are presented on a positive–negative continuum in journalistic coverage of disasters.*

*Theme 2: Journalistic coverage tends to exclude perspectives of older adults from relevant discourse.*

*Theme 3: Journalists assess the value of losses for older adults—“home” as a central concept.*

*Theme 4: Disasters are framed as disrupting retirement ideals.*

### Theme 1: Stereotypes of Older Adults Are Presented on a Positive–Negative Continuum in Journalistic Coverage of Disasters

Both positive and negative stereotypes pertaining to older adults were present throughout disaster media included in this study. Some of the common negative stereotypes included vulnerable, fearful, confused, and scared. In contrast, positive stereotypes included heroic, courageous, and brave. Not all older adults were treated equally when it came to stereotyping; there was a difference between the descriptions applied to “seniors” and “vulnerable seniors.” The term “senior” in many articles was used synonymously with “older adult” and referred to a person of advanced age. Whereas, “vulnerable senior” was used to indicate not only a person of advanced age, but also an individual with a disability—particularly a visible, physical disability such as a mobility impairment. This distinction is reflective of intragenerational ageism, whereby one group of older adults is viewed as unequal or inferior to another group of older adults. In the context of a disaster, membership in either group appeared to elicit compassionate ageism, whereby older adults as a demographic were viewed as victims who need to be taken care of. This implies that advanced age itself is often equated with disability. However, older adults who were also part of the “vulnerable” group were typically described in terms of their visible, physical disability. In the following example, it was noted in the article that Campbell was 84 years old:

“My mom has a had a lot of loss, the family has had a lot of loss,” Dunn said. But Campbell, sitting in her wheelchair in the hospital, took the loss of her home in stride (CBC, 2018 Ottawa- Gatineau Tornadoes).

In this corpus of news media, we identified two examples of factors that influence stereotyping, civic engagement, and culture. Civic engagement by older adults, for example in the form of volunteer work during response and recovery efforts, reduced descriptions consistent with compassionate ageism. Civic engagement led to positive stereotyping and the use of those older adults as symbols of resilience and hope for the greater community. The underlying societal expectation is that older adults will require help from the community, so articles describing older adults giving back appears to counteract this assumption as highlighted by the following example:

What mattered was the community, young and old, near and far, coming together to help each other. There were children filling sandbags and running empty wheelbarrows; there were octogenarians ladling hot soup and handing out water bottles; there were construction guys with trucks filling them full of sandbags… It was wonderful seeing everyone doing what they could do, given their abilities and assets (*The Ottawa Citizen*, 2019 Ottawa-Gatineau Floods).

In terms of culture, Indigenous and non-Indigenous older adults were portrayed distinctly by the media. Generally, Indigenous peoples of advanced age were referred to as “elders,” compared to “older adult” or “senior”; the word “elder” itself denotes authority. Indigenous elders were portrayed as having unique knowledge about the environment because of their cultural and spiritual values. This unique knowledge was then highlighted in partnerships between Indigenous communities, and traditional academic institutions and governments who seek to understand how weather patterns and animal behaviors have changed over time because of climate change. The knowledge of Indigenous elders is valued and seen as an untapped resource as highlighted by the following quotations:

There are relatively few scientific research studies specifically on the James Bay coast. In the absence of western science, traditional Indigenous knowledge - oral histories passed down through generations - provides a vast treasure trove of untapped climate change knowledge (*Toronto Star*, 2019 Ottawa-Gatineau Floods).

Indigenous elders were portrayed by the media as leaders and advocates for their communities and peoples. They were portrayed as being driven by their desire to improve conditions for younger and future generations. Across all five disaster media sources, Indigenous elders were labeled with positive stereotypes particularly with reference to the knowledge they have. The same could not be said for non-Indigenous older adults, whose knowledge was either not discussed or minimized.

Participation in civic engagement and identification of older adults as Indigenous were two example of factors that lead to positive stereotyping by news media in a disaster context. In contrast, negative stereotypes applied to older adults did not appear to follow a “one size fits all approach”; older adults—by virtue of their age—are often viewed as disabled, and the addition of a visible physical disability appears to enhance or further legitimize this belief. Negative, but not positive, stereotypes had a tendency to result in descriptions consistent with compassionate ageism. This leads to different power dynamics whereby power lies with certain groups of older adults and younger demographics.

### Theme 2: Journalistic Coverage Tends to Exclude Perspectives of Older Adults From Relevant Discourse

The resilience of older adults throughout disasters was met with polarizing opinions when explicitly discussed in disaster media. Importantly, the voices of older adults themselves were often missing from this discourse; instead, perceptions of younger demographics were described. Some suggested older adults were resilient as a result of their life experiences, while others suggested that older adults were at a stage in their lives where they are too fragile to deal with such extreme adverse events. The following quotations highlight these conflicting views:

“We have to remember that people who are 65-plus have seen a disaster or two in their lives. Some have seen war, some have lived through a refugee experience, some have seen other trauma,” Braul says. “Our seniors are resilient” (*Calgary Herald*, 2013 Alberta Floods).

Les personnes âgées peuvent réagir psychologiquement plus fortement à des catastrophes naturelles parce qu’elles en sont au stade de leur vie où elles n’ont jamais eu à faire face à de pareils événements [Older adults can react more strongly psychologically to natural disasters because they are at a stage in their life where they may have never had to deal with such events] (Radio-Canada website, 2016 Fort McMurray wildfires).

Discussions of older adults’ capacity to cope with disasters was polarized; however, the actions portrayed by community members toward older adults indicate that vulnerability is both expected and assumed. Indeed, many articles portrayed older adults as not being able to do anything for themselves including getting themselves to safety if needed, maintain a supply of food, and take care of their pets. The following quotations highlight the condescending nature toward the capability of older adults:

Mutchmore and Tessier, both 31, said they realized they couldn’t just sit back and wait for the power to come on because many residents would be confused and scared. “I wanted to make sure everyone was okay,” said Mutchmore. “If you’re sitting in your living room and you have no power, and its pitch black and your ﬂashlight is on the other side of the unit, who knows what you could trip over?” (CBC, 2018 Ottawa-Gatineau Tornadoes).

To appease her, four firefighters will return the next day to extricate Graveline’s dove, Miss Lucy (*The Ottawa Citizen*, 2017 Ottawa-Gatineau Floods).

When older adults’ perceptions were included on topics surrounding resilience and coping, they mentioned strategies including checking in with others, caring for their pets, asking for help when needed, and “focusing on the good.” The opinions presented in the media on the coping capacity of older adults have a tendency to be presented as binary—either resilient or fragile; perceptions of older adults do not appear to inform or influence the discourse. In comparison, actions taken by community members towards older adults are more indicative of compassionate ageism, as vulnerability appears to be both expected and assumed.

### Theme 3: Journalists Assess the Value of Losses for Older Adults—“Home” as a Central Concept

While wildfires, floods, and tornados are different types of disasters, they all elicit fear of loss, which extends beyond loved ones and property to include the potential loss of independence, autonomy, and security. Residing in your own home appears to be a link—particularly later in life—to maintaining this independence, autonomy, and security. Older adults who had their own homes were portrayed as “fighting the good fight,” particularly in the context of flooding and wildfire disasters where action can be taken in the preparation stages to mitigate damages.

After saving a life, there is no greater “emotional rescue” than saving one’s home. Life’s most meaningful moments happen at home. It is most people’s biggest financial risk and many look to the family home as a source of retirement funds (*Montreal Gazette*, 2019 Ottawa-Gatineau Floods).

It is worth noting that discourse on this topic focused on older adults who have or had a home of their own and did not mention those who experienced housing insecurity or homelessness. Without the security of having a home, other retirement ideals are perceived as unattainable and are invisible in this discourse. There appeared to be mixed reaction by older adults to the potential loss of home, while some expressed a sense of loss if their home was damaged, other focused on it as a piece of property that could be replaced.

“I locked the door and I was saying to myself, ‘I may never come back here again.’ It’s heartbreaking because I put my heart and soul into that place trying to make it our retirement home” (*The Ottawa Citizen*, 2018 Ottawa-Gatineau Tornadoes)*.*

“I don’t know, well, it’s only a house and it can be replaced”: 84-year-old woman to rebuild after tornado rips through home (CBC, 2018 Ottawa-Gatineau Tornadoes).

Interestingly, older adults who had to evacuate their homes and rely on social programs or government aid for assistance, and those that lived in a facility such as a retirement home, nursing home, or long-term care were described by other demographics in news media with positive stereotypes like “courageous” and “heroic.” However, in the journalistic coverage of these subgroups, they were more likely to be objectified. For example:

“I don’t know where I am going,” said Michelle Auger, 66, while sitting outside of Gabrielle-Roy campus. “All I know is that they are shipping us out of here today.” (*The Ottawa Citizen*, 2018 Ottawa-Gatineau Tornadoes).

Older adults who had to rely on social programs or government assistance due to evacuation from their own home—or due to the nature of living in a facility—were more likely to be portrayed as passive participants in the disaster response and recovery process. In comparison, older adults who had their own home and the capability or resources to either maintain it or make their own alternate arrangements were more often portrayed as active participants. This speaks to the power dynamics that exist between those who do and do not have their own home and how having a home and/or resources to sustain that home remains a connection to independence, autonomy, and security particularly later in life. Indeed, having your own home later in life promotes Western colonial ideals of retirement such a life of leisure and engagement in philanthropy.

### Theme 4: Disasters Are Framed as Disrupting Retirement Ideals

The concept of “retirement” is a Western colonial ideal promoted as a goal that people should strive to achieve, and is often associated with wealth status indicators such as increased leisure time, travel, and philanthropy. Owning a “dream home” by the water is one such ideal that is portrayed by the media. But with the frequency and severity of disasters increasing due to climate change, older adults impacted by the Ottawa-Gatineau floods in both 2017 and 2019 frequently endorsed not being able to “go through this again” and feeling as through the government was not protecting their interests by continually allowing people to re-build on flood plains. Flooding and wildfires are two such disasters for which there is typically a period of awareness of the impending event and with that comes a desire to place blame or responsibility on government or other agencies for lack of adequate preparedness. In comparison, disasters such as tornados—which often catch people off guard—do not invoke the same tendency in the media to hold someone accountable.

In terms of experiencing multiple floods within a 2-year time span, the decision between taking a government bail-out to move to another area, or use insurance money to re-build, was framed as leaving older adults with both financial and emotional losses. Older adults were cited as thinking about, or returning to work in order to maintain financial stability, as seen in the following quotations:

“I’m retired, so does that mean I have to go back to work to get some money to do [these repairs]?” (CBC, 2019 Ottawa-Gatineau Floods).

Cardinal is a retired public servant, and Briand was too, until the costs of flood repairs altered her plans. She has returned to work in the private sector (*The Ottawa Citizen*, 2019 Ottawa-Gatineau Floods).

The threat of disasters had led to increased concern that older adults will not be able to remain retired or will have to put their retirement plans on hold because of losses sustained. This is further complicated by an aging population and increased numbers of people retiring from the workforce. The Canadian government has not only been criticized for its lack of investment in disaster preparedness but also, its attempts to cut pension plans and social programming, including those for older adults. Fiscal cutbacks in this area speak to a lack of value placed on people who no longer contribute to economic productivity and is an act of intergenerational ageism. Importantly, the discourse surrounding the impact of disasters on retirement did not acknowledge older adults who do not have the financial resources to retire.

### Model: An Overview of the Journalistic Coverage of Older Adults in Disaster Contexts

**Figure 1:**
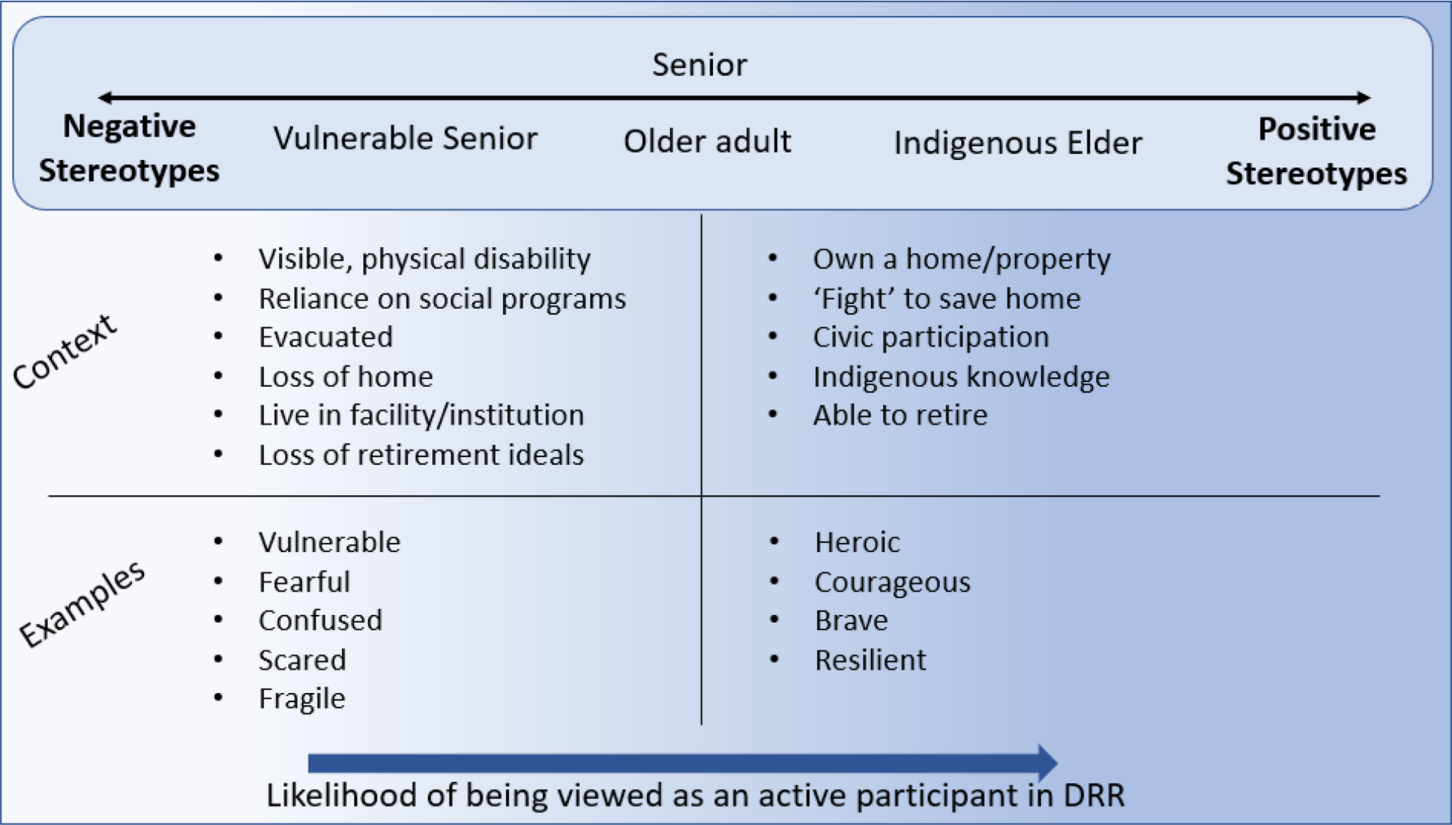
An overview of the journalistic coverage of older adults in disaster contexts. *Note*. DRR = disaster risk reduction.

In this study, stereotypes applied to older adults were conceptualized on a positive–negative continuum (Figure 1). The words “senior” and “older adult” were typically presented as neutral terms to indicate a person of advanced age, while the term “vulnerable senior” lies on the negative end of the spectrum and “Indigenous elder” on the positive end. Below the continuum are contexts in which we found negative and positive stereotypes as well as examples that were applied in journalistic practices. For example, older adults who lost their home because of a disaster were more likely to be negatively stereotyped in news media coverage with descriptors such as vulnerable or fearful. In contrast, older adults who were able to remain in their homes or used resources to “save” their homes were more likely to be labeled with positive stereotypes and described as heroic or courageous.

Interestingly, we found one context in which the stereotype applied to older adults by another demographic in news media coverage, via a quotation, was the opposite of the stereotype applied by the journalist in the same article. Older adults who lived in facilities/institutions or who needed to rely on social services were described positively by another demographic, while the journalistic coverage applied negative stereotypes and descriptors. While only one example of this inconsistency was found, it may highlight discrepancies that exist between the social construction of aging through news media and of the broader societal discourse.

The distinction between different groups of older adults based on the contexts identified is indicative of intragenerational ageism in journalistic practices of disaster-related news media. While the application of negative stereotypes tends to lead to compassionate ageism. This has implications for how older adults are viewed in terms of the DRR process. Older adults labeled with positive stereotypes were more likely to be presented in news media coverage as active participants in the response and recovery phases.

## Discussion

Disasters disproportionately impact older adults and, because of this, they are considered a high-risk population in DRR practices (Intergovernmental Panel on Climate Change [IPCC], 2014; McDermott-Levy et al., 2019). When considering the capabilities of older adults, like other high-risk populations, there is a tendency to focus on deficits rather than assets, and this is exacerbated in a disaster context (O’Sullivan et al., 2014). The media can influence the social construction of resilience and vulnerability, which can further influence DRR practices (Vasterman et al., 2005). Traditionally the media have presented negative depictions of old age and aging, which helps to sustain and perpetuate ageist attitudes, thereby shaping societal discourse (Clarke et al., 2003; Fraser et al., 2020; Rozanova et al., 2006). In this study, we aimed to explore how the Canadian news media portrays older adults and aging in a disaster context.

Examples of compassionate, intra-, and intergenerational ageism were identified in this study. Generally, old age is viewed as a disability that leads to societal stigma; however, older adults who also have a physical and/or cognitive impairment are subjected to a double stigma (Lagacé, Tanguay, et al., 2012). In this study, double stigma was observed to be used as a means of amplifying emotions associated with compassionate ageism. Other predominant examples of ageism found in this study included leaving the voices of older adults out of discourse on coping capacity, expectations of vulnerability and dependence on others, the use of passive, and at times condescending language to describe older adults, and expectations of wealth status indicators denoting traditional ideals of “retirement.” These examples are not unique to news media covering disasters; they have been observed across a broad range of topics covered by the media (Lagacé, Tanguay, et al., 2012; Raynor, 2015). These common examples of ageism contribute to the social construction of vulnerability in old age by influencing perceptions and dominating discourse.

Ageism in a disaster context may be amplified when older adults experience a loss of property such as “home,” or loss of perceived retirement ideals because this can further lead to loss of physical and financial independence, autonomy, and security. Those older adults who were unable to remain in their homes due to a disaster were portrayed similarly in journalistic practices to those who lived in facilities—as passive participants in the response and recovery process. Previous research has argued that a “home” is an extension of the self, a place where a person can relate to society (Laroque, 2011). Loss of home because of a disaster may then have compounding impacts on older adults because, in addition to the aforementioned losses, there is potential to experience a disruption in sense of self, while also being perceived as passive by society further perpetuating ageist attitudes. Importantly, those experiencing homelessness were not mentioned in the corpus of media included in this study, which speaks to power dynamics that exist for those who do and do not have a traditional “home.” It further speaks to the emphasis on physical infrastructure damage in disaster discourse.

Culture appears to influence ageist perceptions in a disaster context. In our study, Indigenous elders were more likely to be the recipients of positive stereotypes when discussing knowledge related to the environment, unlike non-Indigenous older adults. Culture is one of many determinants of the value that society places on old age and aging; in some cultures such as those in Asia, aging is viewed as a progression and a privilege, whereas in others, such as North America, it is viewed as a regression and can lead to stigmatization and social exclusion (Lagacé et al., 2012; O’Reilly, 1998). In western colonial societies, such as Canada, physical and financial independence, economic productivity, and autonomy are highly valued, and these values can legitimize and reinforce ageist perceptions (Cuddy et al., 2005). It is worth noting that there does not appear to be a consensus on whether ageism remains predominantly a Western societal issue, as some studies have suggested that ageism is pervasive and pan-cultural as a result of the modernization of social structures across the globe (Chan et al., 2012; Cuddy et al., 2005; Eyetsemitan et al., 2003).

In this study, a strategy that appeared to mitigate perceptions of ageism was civic engagement. Older adults who participated in volunteer efforts in the response and recovery phases of a disaster were more likely to be labeled with stereotypes and presented as symbols of hope for the community. Indeed, civic engagement by older adults has been suggested as a way to mitigate ageist attitudes in two ways, by allowing older adults a medium through which they can make meaningful contributions to society and by providing opportunities for interactions with other generations (Raynor, 2015). In this way, older adults are perceived not only as consumers of resources but also as providers (Raynor, 2015). Opportunities for older adults to participate in civic engagement falls within an asset-based approach and can support active aging and intergenerational solidarity (Robertson, 2013). Including older adults in civic opportunities appears to support the social construction of resilience.

This research has important implications for DRR practices. By assuming older adults occupy a passive role, ageist perceptions may be viewed as the norm and, as a result, be included in DRR policies and practices. Civic participation is one such strategy that has been suggested to help mitigate ageism in society. Therefore, the inclusion of older adults in discourse on DRR practices may help to mitigate ageist attitudes and present older adults as active participants in this process. Inclusive participation in DRR practices may further contribute to the social construction of resilience of older adults in this context.

By understanding how the media portrays older adults in disasters, we can better understand factors that contribute to their resilience and vulnerability in this context. Efforts should be made by news media not only to include the voices of older adults in this discourse, but to also open it up to include more diverse people such as those experiencing housing insecurity, homelessness, and those who continue to work into old age. Greater media attention focusing on the coping capacity and resilience of older adults could help to counteract the negative perceptions of aging and older adults in society. This can have further implications for policy development because policy makers are often responsive to public opinion (Gollust & Lantz, 2009; Hutchings, 2003).

One of the strengths of this study is that it includes coverage of five different disasters and the corpus of articles included also come from a wide variety of Canadian media sources. However, only online news media articles were included in this study and therefore data from other forms of media such as television and social media were not considered. Future studies should explore how perceptions of older adults and aging will be influenced by the COVID-19 pandemic and how this may impact vulnerability and resilience within this context. Additionally, interventions which promote the inclusive engagement of older adults in DRR practices should also be explored.

## Conclusion

Ageism is a prevalent form of discrimination impacting older adults. In a disaster context, ageist perceptions and attitudes may be amplified by circumstances that threaten the independence, autonomy, and security of older adults. Not all older adults are subjected to the same types or amount of ageism by the media, and this appears to be impacted by culture and perceived level of disability. In a disaster context, the media appear to largely perpetuate the social construction of vulnerability in old age. However, civic engagement by older adults may be one of many ways that ageist perceptions can be mitigated. DRR practices should consider engaging older adults in order to reduce the risk of including ageist attitudes and policies that promote vulnerability in disasters.
